# Long-Term Cold Acclimation Extends Survival Time at 0°C and Modifies the Metabolomic Profiles of the Larvae of the Fruit Fly *Drosophila melanogaster*

**DOI:** 10.1371/journal.pone.0025025

**Published:** 2011-09-21

**Authors:** Vladimír Koštál, Jaroslava Korbelová, Jan Rozsypal, Helena Zahradníčková, Jana Cimlová, Aleš Tomčala, Petr Šimek

**Affiliations:** 1 Institute of Entomology, Biology Centre of the Academy of Sciences of the Czech Republic, České Budějovice, Czech Republic; 2 Faculty of Science, University of South Bohemia, České Budějovice, Czech Republic; University of Dayton, United States of America

## Abstract

**Background:**

*Drosophila melanogaster* is a chill-susceptible insect. Previous studies on this fly focused on *acute* direct chilling injury during cold shock and showed that lower lethal temperature (LLT, approximately −5°C) exhibits relatively low plasticity and that acclimations, both rapid cold hardening (RCH) and long-term cold acclimation, shift the LLT by only a few degrees at the maximum.

**Principal Findings:**

We found that long-term cold acclimation considerably improved cold tolerance in fully grown third-instar larvae of *D. melanogaster*. A comparison of the larvae acclimated at constant 25°C with those acclimated at constant 15°C followed by constant 6°C for 2 d (15°C→6°C) showed that long-term cold acclimation extended the lethal time for 50% of the population (Lt_50_) during exposure to constant 0°C as much as 630-fold (from 0.137 h to 86.658 h). Such marked physiological plasticity in Lt_50_ (in contrast to LLT) suggested that *chronic* indirect chilling injury at 0°C differs from that caused by cold shock. Long-term cold acclimation modified the metabolomic profiles of the larvae. Accumulations of proline (up to 17.7 mM) and trehalose (up to 36.5 mM) were the two most prominent responses. In addition, restructuring of the glycerophospholipid composition of biological membranes was observed. The relative proportion of glycerophosphoethanolamines (especially those with linoleic acid at the *sn*-2 position) increased at the expense of glycerophosphocholines.

**Conclusion:**

Third-instar larvae of *D. melanogaster* improved their cold tolerance in response to long-term cold acclimation and showed metabolic potential for the accumulation of proline and trehalose and for membrane restructuring.

## Introduction

Genus *Drosophila* comprises almost 1500 described species and is thought to be of tropical origin [1]. The ancestral level of cold tolerance in this genus is suggested to be relatively low [2], and most recent species are chill susceptible [3]. This is also true for a common model of modern biology, the fruit fly *Drosophila (Sophophora) melanogaster*. Its pre-adult development halts at temperatures below approximately 10°C [4], [5]. The adults and pupae die when chilled to temperatures below −5°C even for just 2 h, and the larvae are even more chill susceptible [6]. The following are the reasons for studying cold tolerance in this and other such non-cold-hardy organisms. First, the accumulated knowledge about *D. melanogaster* biology makes it a promising model to investigate detailed mechanisms at the suborganismal level. Second, different drosophilid species widely differ in their cold tolerance [7], [8]. For instance, the larva of the temperate/subarctic drosophilid *Chymomyza costata* represents one of the most cold-hardy organisms on the earth as it survives cooling to a temperature as low as that of liquid nitrogen (−196°C) [9], [10]. Third, the level of cold tolerance is a subject of significant phenotypic plasticity [11]–[13]. Thus, both short-term cold hardening and long-term cold acclimation considerably improve cold tolerance of *D. melanogaster* [6], [14]–[17]. Fourth, knowledge on cold tolerance may help in the development of techniques for long-term storage and/or cryopreservation of *Drosophila* strains for research and industry [18].

Previous studies on the cold tolerance of *D. melanogaster* focused mainly on the cold shock responses. Cold shock occurs on rapid cooling without ice formation [19] and induces a specific type of chilling injury (which will be described later). Thus, most previous studies assessed the lower lethal temperatures (LLTs) for relatively brief exposures (hours) to relatively severe cold (subzero temperatures close to the supercooling point [SCP]). In addition, many studies have investigated the influence of rapid cold hardening (RCH) on LLTs and cold shock survival [6], [13]–[16], [20]–[25]. RCH is a quick cold acclimation response that occurs within minutes to hours of pre-exposure to a non-lethal low temperature and improves survival after subsequent cold shock [26]. Cold shock response is just one aspect of cold tolerance, however. The injuries caused by low temperatures are likely numerous, heterogenous, and complex. The three basic types of cold injury generally distinguished [27] are *freezing* of body water, *direct chilling* injury, and *indirect chilling* injury. *Freezing* of body water may result in mechanical damage to delicate intracellular ultrastructures and the extracellular matrix. It also causes cellular dehydration and freeze concentration of solutes, likely causing them to reach toxic concentration levels [28]. *Direct chilling* injury results from cold shock, which may cause dissociation of multimeric macromolecular complexes, protein denaturation [29], [30], membrane lipid phase transitions, massive ion leakage, and cell death [31], [32]. *Indirect chilling* injuries accumulate over relatively long exposures (days to months) to relatively mild cold (temperatures well above the SCP, often around or above zero). Disturbed coordination of various metabolic pathways (disorder), excess production of reactive molecules (oxidative stress), disturbance of ion homeostasis, or depletion of available free chemical energy (basically adenosine triphosphate [ATP]) are the most likely causes of indirect chilling injury [33]–[37].

In contrast to the wealth of studies on cold shock responses of *D. melanogaster*, our knowledge on how it copes with indirect chilling injuries and what is the role of long-term cold acclimation is considerably less. Previous studies have indicated that the ability of the third-instar larvae of *D. melanogaster* to survive at 0°C is very low. For instance, a study [6] reported less than 20% survival after 2-h-long exposure to 0°C. It is believed that long-term cold acclimation in insects is based, at least partly, on the accumulation of cryoprotective solutes and restructuring of biological membranes [27], [38]–[40]. Cold acclimation of the larvae of *C. costata* is associated with extensive remodeling of the lipid composition of their membranes [41] and with the accumulation of high amounts of proline, which is directly associated with the ability to survive freezing and cryopreservation in liquid nitrogen [10], [42].

The main objective of this study was to examine the influence of long-term cold acclimation on the development of indirect chilling injuries (mortality) in the third-instar larvae of *D. melanogaster*. The larvae were acclimated by rearing at different constant temperatures of 25°C, 15°C, and 15°C followed by 6°C. Survival tests were performed at constant 0°C. Third-instar larvae were selected in order to obtain the comparative data for *C. costata*, which attains its maximum cold tolerance (survival in liquid nitrogen) in this developmental stage [10]. We tested two related hypotheses that long-term cold acclimation of *D. melanogaster* larvae (a) improves their cold tolerance (extends Lt_50_ at 0°C) and leads to the development of the ability to survive freezing injury and (b) modifies the metabolomic profiles of organic acids, free amino acids, free fatty acids, sugars, and polyols and stimulates restructuring of biological membranes.

## Methods

### Insect rearing and acclimation

The laboratory stock of fruit flies, *Drosophila (Sophophora) melanogaster* (Meigen, 1830), strain Oregon, was maintained at constant 18°C with 12-h/12-h light/dark (L/D) cycle on a diet containing agar (1%), sugar (5%), yeast (4%), cornmeal (8%), and methylparaben (0.2%). For experiments, approximately 30 female flies that were 5- to 10-d-old were allowed to lay eggs in a vial (40 mL) containing 5 mL of standard diet, for 24 h (one L/D cycle). Thereafter, the flies were removed from the vial, and the embryos and larvae developing in the diet were raised under the following three different acclimation treatment conditions.

- 25°C acclimation—rearing at constant 25°C with 12-h/12-h L/D cycle for 5 d
- 15°C acclimation—rearing at constant 15°C with 12-h/12-h L/D cycle for 14 d
- 15°C→6°C acclimation—rearing at constant 15°C with 12-h/12-h L/D cycle for 14 d, followed by rearing at constant 6°C and continuous darkness for 2 d.

We sampled pre-wandering larvae of similar physiological age (but different ages in days) in all the treatments. At the end of an acclimation treatment, when the first wandering larvae occurred, the largest specimens of third-instar larvae were collected from the diet and subjected to survival experiments or processed for biochemical analyses. To avoid thermal stress, the larvae were washed out of the diet by using water of the same temperature as that in the respective treatment.

### Survival at low temperatures

For survival experiments, groups of approximately 20 pre-wandering larvae were placed in 1 g of larval diet in a plastic tube (diameter, 1 cm; length, 5 cm), which was plugged with artificial cotton. The larvae were then exposed to either (a) constant 5°C or 6°C (maintained in a programmable thermostat, F32-ME; Julabo, Seelbach, Germany); or (b) constant 0°C (maintained in melting ice); or (c) a temperature program (set in the programmable thermostat F32-ME in combination with a temperature probe, TC-08; Pico Technology, St. Neots, United Kingdom). The temperature program started at −1°C and comprised three steps: (i) cooling to −2°C at a rate of 0.033°C min^−1^ (30 min); (ii) cooling to −5°C at a rate of 0.1°C min^−1^ (30 min); and (iii) heating to +5°C at a rate of 0.33°C min^−1^ (30 min). Thus, the larvae spent a total of 75 min at subzero temperatures. At the start of the program, a small ice crystal was either added on the surface of diet (freezing condition) or not added (supercooling condition). Adding the ice crystal resulted in an almost immediate freezing of water in the diet and, probably, ice inoculation and freezing of larval body fluids. When no ice was added, the diet did not freeze (no freeze exotherm observed), and the larvae probably supercooled. After cold exposure, the tubes with the larvae were kept at 18°C with 12-h/12-h L/D cycle for 1 week, and pupariation was scored as a criterion of survival.

### Physiological parameters

Individual fresh mass (FM) of 20 larvae in each acclimation treatment was measured using a Sartorius electronic balance (precision, 0.01 mg). The weighed specimens were dried at 60°C for 3 d, and their dry mass (DM) was measured. Water mass (WM, in mg) and hydration (H, in mg; water·mg^−1^ DM) were calculated from the gravimetric data.

A total of 5 larvae in 4 replications were processed from each acclimation treatment to determine the basic biochemical parameters. Total proteins were measured by the bicinchoninic acid protein assay [43] after extraction of total water-soluble proteins by using 50 mM Tris, pH 6.8, followed by re-extraction of detergent-soluble proteins from a centrifugation pellet in the same buffer by the addition of 0.5% deoxycholate and 0.1% sodium dodecyl sulfate (SDS). Total lipid content was measured by spectrophotometric analysis with phosphoric acid-vanillin solution [44] after extraction of lipids by using chloroform∶methanol solution (2∶1, v/v) [41]. Glycogen content was measured by colorimetric determination by using phenol and concentrated sulfuric acid [45] after extraction of glycogen in hot alkali [46].

SCP refers to the temperature at which spontaneous freezing of body water occurs during gradual cooling of insect specimen. A constant cooling rate of 0.2°C min^−1^ was used and the minimum temperature was set to −30°C. The individual larvae were attached to thermocouples (type K, Pico Technology) using thermally conductive paste Cooler Master HTK-002 (Sundial Micro, Ontario, CA, USA).We measured the SCP of 16 larvae in each acclimation treatment by recording the exotherms associated with water-ice phase transition, using the programmable thermostat F32-ME (Julabo) in combination with the temperature data logger TC-08 (Pico Technology).

### Metabolomic profiling

The metabolomic profiles were extensively investigated by a set of targeted and nontargeted mass spectrometry (MS)-based analytical methods. Whole larvae (10 larvae in 4 replications from each acclimation treatment) were homogenized and extracted in 70% ethanol. Low-molecular-weight sugars and polyols were quantitatively determined in the ethanolic extracts after *o*-methyloxime trimethylsilyl derivatization and subsequent analysis by gas chromatography (GC) coupled to MS (GC/MS), as described previously [47]. Nontargeted metabolomic profiling was done by performing a combination of GC/MS and liquid chromatography (LC) coupled to MS (LC/MS) in the same ethanolic extracts after their treatment with ethyl chloroformate under pyridine catalysis and simultaneous extraction in chloroform [48], [49].

GC/MS metabolite profiles were obtained on a VF-17 capillary column (Agilent, Santa Clara, CA, USA) coupled to a dual-stage quadrupole (DSQ) mass spectrometer (Thermo Fisher Scientific, San Jose, CA, USA) equipped with an electron impaction ion source and operated in the full-scan mode from 40 to 500 amu. A Thermo Trace gas chromatograph with a programmable injector and interface hold at 230°C was directly coupled to the mass spectrometer via an interface held at 250°C. A 0.5-µL aliquot of the chloroform extract was injected in the splitless mode into the GC/MS column. Oven temperature was initially maintained at 50°C for 1 min. Thereafter, it was raised to 302°C at a rate of 12°C min^−1^ and maintained for 2 min. Helium was used as the carrier gas and delivered at a constant flow rate of 1.2 mL min^−1^.

LC/MS metabolite profiles were measured after evaporating a 30-µL aliquot of the chloroform extract to dryness by using a mild stream of nitrogen. After dissolution in 200 µL of the LC mobile phase, a 5-µL aliquot was injected into and separated on a Kinetex C18 column (150×2 mm; internal diameter [ID], 2.6 µm; Phenomenex, Torrance, CA, USA) at 35°C at a flow rate of 200 µL min^−1^, using a gradient elution with the mobile phase consisting of (A) 5 mM ammonium formate in methanol and (B) 5 mM ammonium formate in water. The gradient elution program was linear from 30% to 100% A for 12 min, then held at 100% A for14 min, and finally equilibrated for 5 min.

The nontargeted GC/MS and LC/MS data were processed with the Thermo Scientific Xcalibur 2.1 software and an in-house developed Metabolite Mapper platform, which provides automated peak detection and metabolite deconvolution by employing retention time and mass spectral and detector response features, followed by time alignment of the data obtained in each particular analysis for a defined experimental sample set and generation of data matrix, which is automatically exported to a predefined Microsoft Excel™ spreadsheet for further statistical processing. The 39 major metabolites were identified against relevant standards and further subjected to quantitative analysis by using an internal standard calibration method. All chemicals used were purchased from Sigma-Aldrich Co. (St. Louis, MO, USA), except the isotope-labeled metabolites used as internal calibration standards, which were obtained from Cambridge Isotope Laboratories (Andover, MA, USA). Whole-body concentrations of the metabolites were recalculated as mmol·L^−1^ of whole body water (mM).

### Glycerophospholipids (GPLs)

Whole larvae (5 larvae in 8 replications from each acclimation treatment) were homogenized and extracted in ice-cold chloroform∶methanol solution (2∶1, v/v) by using a previously described method [50], [51]. After extraction, the solvents were evaporated under a stream of nitrogen, and lipids were stored at −80°C until analysis. High-performance LC (HPLC) combined with electrospray ionization MS (ESI-MS) [17], [52] was performed on an LC quadrupole (LCQ) ion-trap mass spectrometer (Thermo Fisher Scientific) coupled to a Rheos 2000 ternary HPLC system (Flux Instruments, Basel, Switzerland) equipped with a FAMOS autosampler and Thermos thermostat. The stored dry samples were dissolved in 1 mL of methanol, and 5-µL aliquots were injected into a Synergi Polar HPLC column (150×2 mm; ID, 3.5 µm; Phenomenex). The mobile phase consisted of (A) 10 mM ammonium acetate in methanol, (B) 10 mM ammonium acetate in water, and (C) isopropanol. A linear gradient of A∶B∶C changing from 90∶10∶0 to 70∶0∶30 within 14 min was applied with a flow rate of 300 µL min^−1^. The column temperature was maintained at 30°C. The mass spectrometer was operated either in the positive or the negative ion detection mode at +4 kV or −3.6 kV, respectively. Capillary temperature was 240°C, and nitrogen was used as both the sheath and the auxiliary gas. For MS2 and MS3 fragmentations, ion isolation windows were 5 Da and 2 Da, respectively. The maximum ion injection time was 100 ms; collision energies were 30% (MS2) or 35% (MS3); and mass range of 600 Da to 800 Da was scanned every 0.5 s. The basic chemicals used for extraction and analysis were purchased from Sigma-Aldrich Co. Calibration standards were obtained from Avanti Polar Lipids (Alabaster, AL, USA).

The GPL analysis results were expressed in relative values, *i.e.*, relative proportion of each GPL species from the total of 100%. Our analysis was focused on the major glycerophosphoethanolamines (GPEtns), glycerophosphocholines (GPChols), and glycerophosphoserines (GPSers). The relative proportions of individual fatty acyls (FAs) were calculated from the GPL data.

### Statistical analyses

Survival data (proportions of pupariated larvae) were fitted to exponential decay curves, using the following formula.
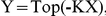
where Top is survival in a control (untreated) group of larvae and K denotes the slope of decay. The K parameters in the different acclimation treatments were compared using the F-test. The time of exposure to low temperature that was lethal for 50% of the larvae in a sample (Lt_50_) was calculated from the exponential curves.

One-way analysis of variance (ANOVA) tests were used to analyze whether the acclimation treatments influenced the physiological and biochemical parameters. Bonferroni post-hoc tests were applied to identify the differences among the acclimation treatments. The analyses were performed using Prism v. 4 (GraphPad Software, San Diego, CA, USA).

The complex association of metabolomic changes with acclimation treatments was determined by principal component analysis (PCA) with Canoco v. 4.52 for Windows (Biometris-Plant Research International, Wageningen, The Netherlands).

## Results

### Long-term cold acclimation significantly improved survival at low temperatures

**Figure 1:**
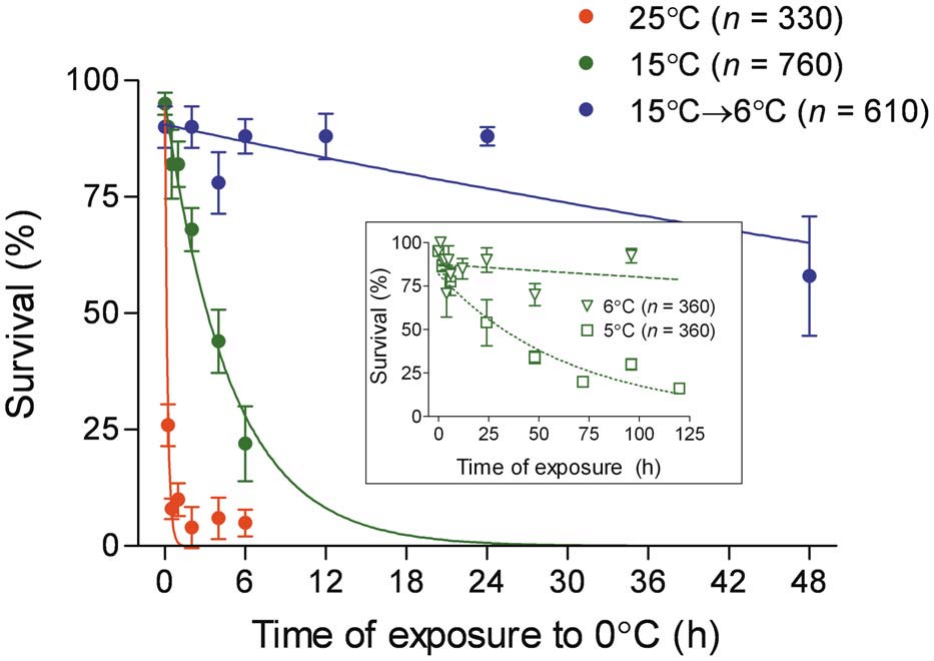
Survival at low temperatures in variously acclimated third-instar larvae of *Drosophila melanogaster*. The three acclimation treatments (25°C, 15°C, and 15°C→6°C) are explained in the text. Each point shows the mean ± standard error of mean (SEM) of survival at 0°C in a group of approximately 20 larvae (replications, 3–8; total, *n*). Survival data were fitted to exponential decay curves. The inset shows the survival of 15°C-acclimated larvae at two different temperatures, 6°C and 5°C.

We observed a clear acclimation effect of relatively low rearing temperature of 15°C on subsequent survival at low temperatures in the third-instar larvae of *D. melanogaster*. While Lt_50_ at 0°C was as short as 0.137 h (8 min 13 s) for the larvae acclimated at 25°C, it increased to 3.165 h (3 h 9 min 54 s) for those acclimated at 15°C. Survival of the 15°C-acclimated larvae further improved on exposure to 6°C for 2 d. After this treatment, Lt_50_ at 0°C increased to 86.658 h (3 d 14 h 39 min 29 s) (Fig. 1). The K parameters of the survival curves differed significantly among the three acclimation treatments (F-test: *F* = 154.0, degrees of freedom of the numerator [dfN] = 2, degrees of freedom of the denominator [dfD] = 15; *P*<0.0001). We used the temperature of 6°C for the acclimation treatment because this was the lowest temperature at which no significant mortality was observed within the 2 d-long exposure of the 15°C-acclimated larvae. In contrast, Lt_50_ was as short as 32.356 h at 5°C (Fig. 1, inset).

The larvae did not show survival ability in conditions favorable for external ice inoculation and partial freezing of their body fluids. None of the larvae survived cooling to −5°C when freezing of the surrounding diet was stimulated by adding a small ice crystal. In contrast, relatively high proportions of the larvae survived cooling to −5°C under the supercooling condition. However, acclimation at 15°C or 15°C→6°C was a prerequisite for their survival in supercooled state (Table 1).

**Table 1:** Survival of variously acclimated 3^rd^ instar larvae of *Drosophila melanogaster* on freezing/supercooling to −5°C.

Acclimation treatment	Cooling condition#	Total (*n*)	Survival (%)
25°C	freezing	30	0
	supercooling	31	0
15°C	freezing	57	0
	supercooling	49	38.6
15°C→6°C	freezing	38	0
	supercooling	48	50.0

# See text for detailed description of the temperature program; larvae were cooled in their standard diet and freezing was stimulated by adding a small ice crystal on the diet surface.

### Physiological and biochemical changes associated with cold acclimation

Although the physiological ages of the larvae reared at 25°C and those reared at 15°C were similar (the FM of the larvae was consistently within 85%–95% of the “final” FM of the wandering larvae in the respective treatment), the latter grew bigger and had larger reserves of total lipids than the former (see Dataset S1 for detailed results and statistical analysis). Hydration, total protein content, and glycogen levels were similar in the larvae in the two acclimation treatments. Further acclimation of the 15°C-reared larvae at 6°C for 2 d resulted in significant reduction in FM, DM, and glycogen levels, while hydration, total protein content, and total lipid content remained constant. SCPs of the larvae in all the three acclimation treatments were similar and relatively low (means ranged between −19.9°C and −20.2°C; Dataset S1).

**Figure 2:**
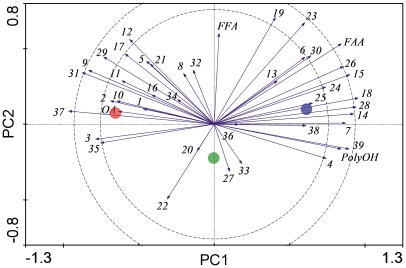
Principal component analysis showing the association between acclimation treatments (points) and concentrations of metabolites (eigenvectors) in the third-instar larvae of *Drosophila melanogaster*. Red point, 25°C; green point, 15°C; blue point, 15°C→6°C. The numbers (metabolites) are partially decoded in the text and completely listed in Dataset S2. PC1 axis explains 47.0% of the variation and PC2 axis explains additional 31.5% of the variation (Monte Carlo test: *F* = 20.508; *P* = 0.0010). The eigenvectors extending beyond the inner and outer dashed circles represent the compounds that fit the model by more than 60% and 90%, respectively. OA organic acids; FFA, free fatty acids; FAA, free amino acids; PolyOH, sugars and polyols.

**Figure 3:**
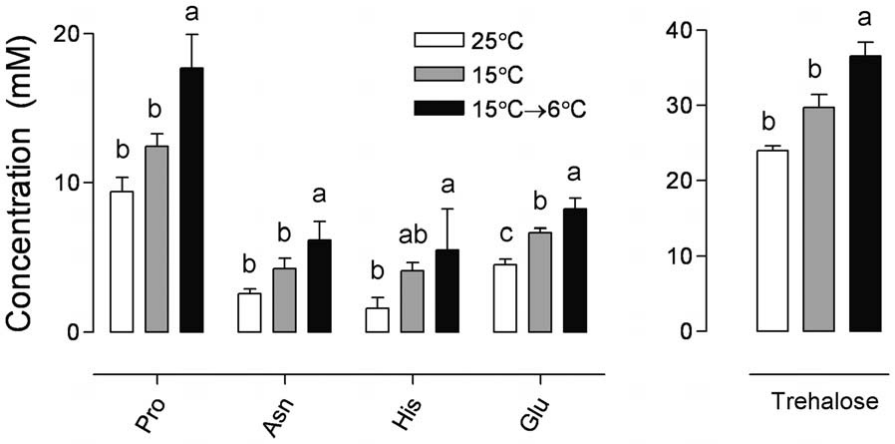
Acclimation-related changes in selected metabolites in the third-instar larvae of *Drosophila melanogaster*. Each column represents the mean ± standard deviation (SD) of 4 independent replications (10 larvae each). Influence of acclimation treatment on metabolite concentration was tested by ANOVA followed by Bonferroni post-hoc test (means indicated with different letters are significantly different). Pro, proline; Asn, asparagine; His, histidine; Glu, glutamate.

We investigated the changes in the profiles of 39 major metabolites that were present in detectable amounts in most samples and the identities of which were verified by MS. The concentrations of approximately two-thirds of the metabolites were significantly influenced by the acclimation treatments (see Dataset S2 for a complete list of metabolites and statistical analyses). PCA identified a group of metabolites that showed an increase in concentration during the cold acclimation process (Fig. 2). This group included citrate (no. 4), alpha-aminobutyric acid (no. 7), proline (no. 14), asparagine (no. 15), glutamate (no. 18), tryptophan (no. 26), putrescine (no. 28), and trehalose (no. 39) (the numbers in parentheses correspond to the numbers shown in Fig. 2; more details in Dataset S2). The details of the five most abundant metabolites in this group are shown in Fig. 3. The sum concentration of sugars plus polyols and that of free amino acids increased during cold acclimation, while the sum concentration of detectable organic acids decreased. The sum concentration of free fatty acids remained unchanged (Fig. 2).

**Figure 4:**
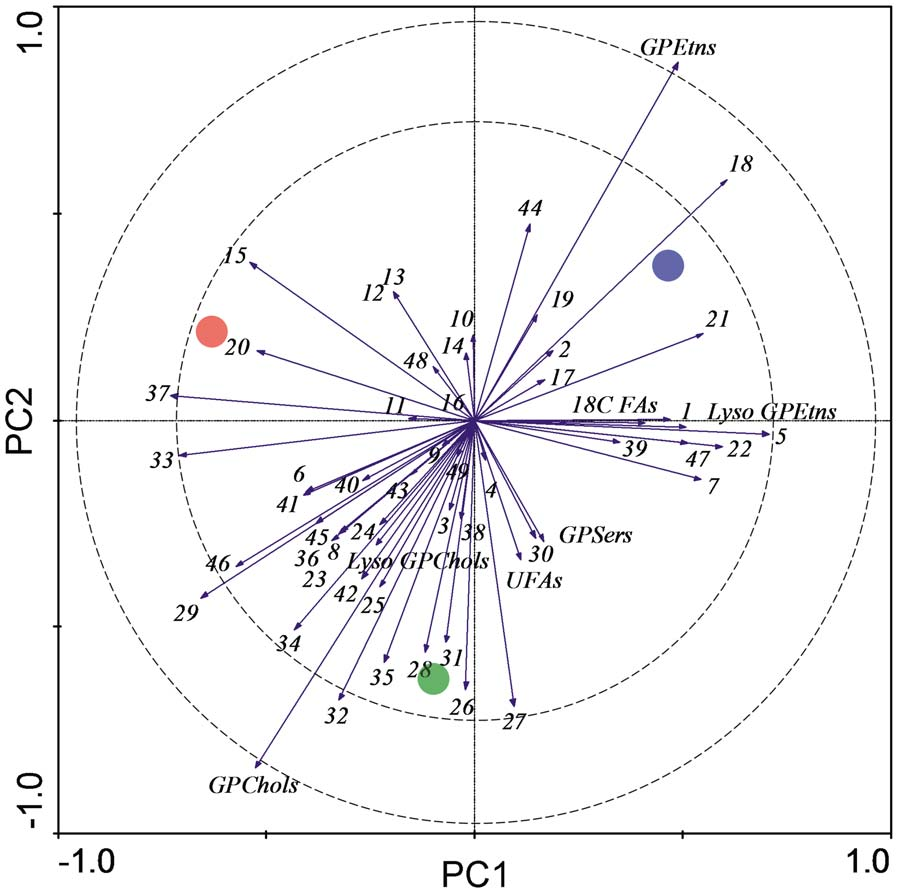
Principal component analysis showing the association between acclimation treatments (points) and relative proportions of glycerophospholipids (eigenvectors) in the third-instar larvae of *Drosophila melanogaster*. Red point, 25°C; green point, 15°C; blue point, 15°C→6°C. The numbers (glycerophospholipids) are partially decoded in the text and completely listed in Dataset S3. PC1 axis explains 32.3% of the variation and PC2 axis explains additional 31.3% of the variation (Monte Carlo test: *F* = 8.471; *P* = 0.0020). The eigenvectors extending beyond the inner and outer dashed circles represent the compounds that fit the model by more than 60% and 90%, respectively. GPEtns, glycerophosphoethanolamines; GPChols, glycerophosphocholines; GPSers, glycerophosphoserines; UFAs, unsaturated fatty acyls; 18C FAs, 18-carbon fatty acyls.

**Figure 5:**
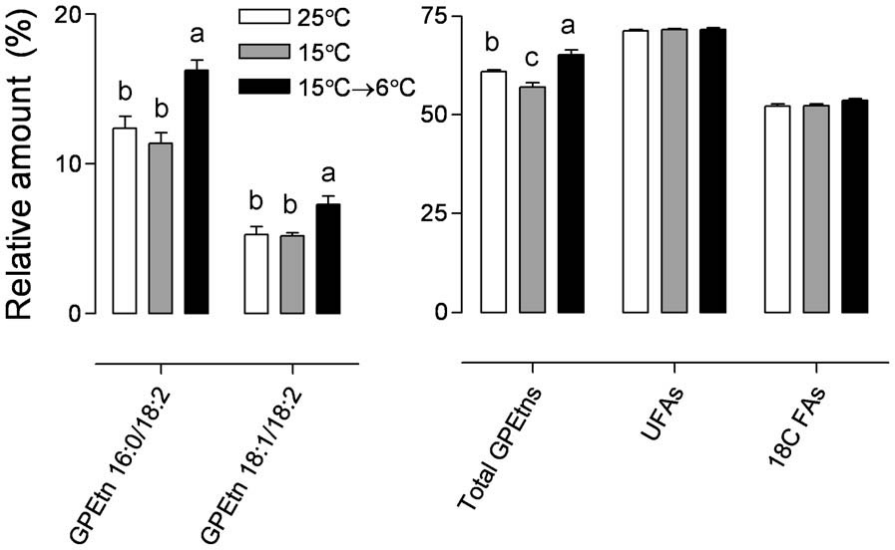
Acclimation-related changes in selected glycerophospholipids and calculated parameters in the third-instar larvae of *Drosophila melanogaster*. Each column represents the mean ± SD of 4 independent replications (5 larvae each). The influence of acclimation treatment on relative proportions was tested by ANOVA, followed by Bonferroni post-hoc test when significant influence was detected (means indicated with different letters are significantly different). GPEtn 16∶0/18∶2, 1-palmityl-2-linoleyl-*sn*-glycerophosphoethanolamine; GPEtn 18∶1/18∶2, 1-oleyl-2-linoleyl-*sn*-glycerophosphoethanolamine; UFAs, unsaturated fatty acyls; 18C FAs, 18-carbon fatty acyls.

We identified 41 different GPLs (list and statistical analysis in Dataset S3). Most of the GPL species were present in relatively small proportions, not exceeding 1% of the total GPL pool. Almost 50% of the GPL species exhibited statistically significant acclimation-related changes (Dataset S3). PCA identified two GPEtns, GPEtn 16∶0/18∶2 (no. 18) and GPEtn 18∶1/18∶2 (no. 21), which showed a close association with the most cold-hardy group of larvae (Fig. 4). Detailed results for these two compounds are shown in Fig. 5, which also illustrates some of the changes in parameters calculated from the GPL composition data. The relative proportion of total GPEtns was slightly lower in the 15°C-acclimated larvae than in the 25°C-acclimated larvae, but it significantly increased (from 57.0% to 65.2%) in the 15°C→6°C-acclimated larvae. The relative proportions of total unsaturated FAs and total 18-carbon FAs did not change significantly with cold acclimation (Fig. 5).

## Discussion

We found that long-term cold acclimation considerably improved cold tolerance in the third-instar larvae of *D. melanogaster*. A comparison of the larvae acclimated at constant 25°C with those acclimated at constant 15°C followed by constant 6°C for 2 d (15°C→6°C) showed that long-term cold acclimation extended the survival time (or Lt_50_) at constant 0°C as much as 630-fold (from 0.137 h to 86.658 h) (Fig. 1). Our data indicate that LLT was also shifted by cold acclimation, although this was not focused upon in our study. About 50% of the larvae survived supercooling to −5°C for a brief period when acclimated at 15°C→6°C, while none of the larvae survived the same treatment when acclimated at 25°C (Table 1).

### Nature of cold injury and effect of cold acclimation

It is not easy to directly compare our results with those of other studies on cold tolerance of *D. melanogaster* because, as pointed earlier [11], various authors have used at least 27 different tests of cold tolerance and assessed different metrics of response (survival, chill coma onset or recovery, reproductive success). Most previous studies have dealt with cold shock situation, and one clear commonality can be derived: LLT is close to −5°C and is *relatively fixed* [6], [12]–[17], [25]. This means that relatively low variability or plasticity, spanning a range of only a few degrees, has been observed for LLT at the evolutionary or physiological level, respectively. Thus, LLT is similar in closely related *Drosophila* species [2] in different populations of *D. melanogaster* [15], [53] and in different generations of experiments on the selection for cold shock tolerance (low adaptive evolutionary variability) [14], [25], [54]. Similarly, both RCH and long-term cold acclimation have shown relatively weak effects on LLT, shifting it by only a few degrees at the most (low physiological plasticity associated with acclimation) [15]–[17].

In contrast to the relatively fixed LLT, Lt_50_ at 0°C has shown dramatic plasticity in response to long-term cold acclimation in *D. melanogaster* larvae (this study) and adults [17]. LLT suitably describes the effect of cold shock, while Lt_50_ is much better suited for describing the effect of indirect chilling injury. This is because the physiological bases of cold shock injury and indirect chilling injury are likely different [16], [27], [55], [56]. *Acute* direct chilling injury during cold shock is probably related mainly to protein denaturation and membrane lipid phase transitions [29]–[32]. Acclimation mechanisms (associated with both RCH and long-term cold acclimation) apparently have only a limited ability to prevent/repair this type of injury, *i.e.*, to shift the LLT by more than a few degrees. On the other hand, *chronic* (cumulative) indirect chilling injury is probably related to the inability to maintain homeostatic processes, which results in a lack of free chemical energy, metabolic disorder, oxidative stress, and disturbance of ion homeostasis [33]–[37]. The deleterious effects of this type of injury can relatively be well prevented by acclimation processes, especially long-term cold acclimation. This is why Lt_50_ shows such marked plasticity. Long-term cold acclimation involves numerous adjustment processes that require considerable time to be executed properly, such as gradual cessation of cold-sensitive processes (cell cycle, morphogenesis, development, reproduction) [57], regulated metabolic suppression [33], bolstering the antioxidative potential [34], accumulation of cryoprotective solutes [39], synthesis of specific proteins with antifreeze or ice nucleation activity [58]–[60], membrane restructuring [40], stimulation of protein stabilization/refolding machinery [61], [62], and channel arrest [33], [35]. In contrast, the physiological nature of acclimation processes beyond RCH remains largely unknown, but these processes may involve accumulation of cryoprotective solutes such as glycerol, rapid membrane restructuring, and stimulation of repair mechanisms [17], [25], [26].

### Meanings of upper limit of cold injury zone and SCP

Theoretical modeling and fitting of experimental data for various insects have revealed that an *upper* threshold temperature may exist for indirect chilling injury. The lowest temperature that causes no significant mortality during prolonged exposure to cold is referred to as upper limit of cold injury zone (ULCIZ) [63], [64]. We did not collect sufficient data in our study to estimate the value of ULCIZ precisely. The Lt_50_ of the 15°C-acclimated *D. melanogaster* larvae was 3.2 h at 0°C, extended to 32.4 h at 5°C, and further to >48 h at 6°C (Fig. 1). We suppose, however, that cold-related mortality occurs even above 6°C. The lower threshold temperature for larval development (LDT) is approximately 10°C [4], [5]. Hence, at temperatures below 10°C, the larvae cannot continue to develop and become fated to die. If we accept that the main cause of their death is the inability to maintain the balance between energy demand and supply and thus support other homeostatic functions that depend on access to free energy, then the death is due to accumulation of typical indirect chilling injuries. This suggests that ULCIZ and LDT are, in fact, the same thresholds in *D. melanogaster* larvae. This may be a rule for other chill-susceptible, quiescent insects. On the other hand, the concept of ULCIZ is useful for chill-tolerant, diapausing insects. Rich empirical knowledge of many researchers shows that these insects can be stored for very long periods (months to years) at low temperatures (typically around 0°C) without much loss of viability, *i.e.*, above ULCIZ [62].

Is it theoretically possible to identify a *lower* threshold temperature for indirect chilling injury, analogous to ULCIZ? In experimental setting, it would mean to identify a threshold temperature at which acute direct chilling injuries start to occur and prevail over the chronic effects of indirect chilling injuries. Perhaps, −2°C can be close to such a threshold temperature for *D. melanogaster*, as some experiments have indicated that survival of various developmental stages rapidly declines when exposure temperature drops below −2°C [13], [15].

Another threshold temperature, SCP, can be measured easily and precisely. In our experiments, it was close to −20°C in all the acclimation treatments. The ecophysiological meaning of SCP, however, is limited in *D. melanogaster* larvae, as in the other chill-susceptible insects [3]. SCP represents the temperature at which ice crystallization of body fluids occurs when larvae are in a dry environment, *i.e.*, without any surrounding ice. External ice crystals may stimulate freezing of body fluids at temperatures close to 0°C (inoculation by external ice). In fact, such inoculation is a prerequisite of freeze tolerance in the larvae of *C. costata* and some other insects [42]. Therefore, we tested whether our acclimation treatments could stimulate development of freeze tolerance in *D. melanogaster* larvae. None of the larvae survived in our freezing tests despite that the conditions were relatively mild (slow cooling and melting rates, inoculation by external ice at −1°C, minimum temperature of −5°C, and total 75 min spent at subzero temperatures) (Table 1). We have no direct evidence that the mortality observed in the freezing tests was indeed caused by freezing injuries. Nevertheless, we consider it highly probable because the larvae survived the same temperature program under the supercooling condition (*i.e.*, without ice nucleation). Our data are supported by previous studies, in which no ability to tolerate freezing injury has been observed in the larvae of 22 different species of *Drosophila* [2].

### Metabolomic profiling and long-term cold acclimation

Almost two-thirds of the 39 major metabolites identified in this study exhibited statistically significant concentration changes in response to long-term cold acclimation. Most of the changes, however, were relatively small and rarely reached a several-fold magnitude. For instance, the concentration of putrescine in the 15°C→6°C-acclimated larvae was approximately 4.5-fold that in the 25°C-acclimated larvae. This difference was approximately 3-fold for the concentrations of the amino acids asparagine and histidine. Most metabolites were present in relatively low concentrations (<10 mM), which makes their effective contribution to cryoprotective functions unlikely.

Two compounds, trehalose and proline, were present in relatively high amounts (>10 mM) and showed a positive association with increasing cold acclimation (Figs. 2 and 3). Both these compounds have received much attention in previous studies as they belong to a group of compatible solutes, *i.e.*, they are accumulated in relatively high concentrations in variously stressed organisms and play different protective roles in these organisms [65]–[69]. Previous studies have observed accumulations up to several hundred mmol·L^−1^ in many overwintering insects, for both trehalose [39] and proline [70]–[73]. When accumulated in relatively high amounts (which is not the case of *D. melanogaster* larvae), these compounds can colligatively contribute to the extension and stabilization of supercooling [74] or can stimulate vitrification, *i.e.*, transition of body water from the liquid phase to an amorphous, glass-like phase during drying or freezing [75]–[77]. At relatively low concentrations, they may non-colligatively protect the native macromolecular structures such as proteins and biological membranes by preferential exclusion from their hydration shells [78], [79]. In addition to such non-specific cryoprotective mechanisms, some specific roles have been attributed to trehalose and proline. Trehalose can assist refolding of unfolded proteins by molecular chaperones [80], [81]; serve as a scavenger of oxygen radicals [82]; and directly replace missing water molecules in the hydration shells of proteins and phospholipid membranes during desiccation [83]–[85]. Amphipathic proline molecules can intercalate between the headgroups of membrane phospholipids during freeze dehydration and alleviate mechanical stresses in the membranes or can disturb the membranes, making them less prone to the liquid crystalline-to-gel transition [65].

In comparison to other cold-acclimated insects, the larvae of *D. melanogaster* in our study showed relatively low levels of accumulated trehalose and proline. We therefore consider it premature to speculate whether these compounds causally contributed to cold tolerance of the larvae and/or which of the abovementioned mechanisms was involved. Nevertheless, three aspects of our metabolomic analysis are quite interesting when compared with the literature data. First, several previous studies have investigated RCH-induced changes in putative cryoprotectants in *D. melanogaster* adults and obtained mixed results. Targeted analysis of glycerol did not confirm glycerol accumulation [23], [24]; nontargeted proton nuclear magnetic resonance (^1^H-NMR) metabolomic profiling showed increase in trehalose and glucose levels and no change in proline levels [86]; and targeted analysis of glucose failed to confirm glucose accumulation in a slightly modified RCH protocol [25]. These observations indicate that (a) metabolomic profiles are sensitive to small modifications in acclimation conditions, which themselves have little or no effect on cold tolerance and (b) some facets of acclimation response are shared between RCH and long-term cold acclimation (such as trehalose accumulation), while some others differ (such as proline accumulation occurring only during long-term cold acclimation). Second, in a previous study, a 3- to 6-fold increase was found in the proline levels in *D. melanogaster* strains selected for resistance to chilling injury at 0°C for 30–60 h or to cold shock at −7°C for 2–3 h [87]. This observation supports our results, suggesting that there is a close association between proline level and cold tolerance. Third, trehalose and proline are two compounds that exhibit the clearest accumulation responses to diapause transition and long-term cold acclimation in *C. costata*, reaching the levels of 56 mM (trehalose) and 147 mM (proline) in diapausing cold-acclimated larvae [10], [42]. In addition, a direct evidence of the essential role of proline in high freeze tolerance, including survival in liquid nitrogen, has been obtained by artificially increasing the levels of proline in the tissues of *C. costata* larvae [10]. Thus, the larvae of *D. melanogaster* possess the metabolic ability to accumulate proline, the same as that critical for the development of high cold tolerance in the larvae of the related species *C. costata*. These results open avenues for further investigating the role of proline in the cold tolerance of *D. melanogaster*.

### Membrane restructuring in response to long-term cold acclimation

Long-term cold acclimation stimulated small but statistically significant changes in the lipid composition of biological membranes in *D. melanogaster* larvae (Figs. 4 and 5). Membrane restructuring in response to cold has been documented in various poikilotherms, including insects. Several basic patterns have been repeatedly reported such as FA desaturation, shortening of average FA chain length, increase in the relative proportion of GPEtns, and reshuffling of FAs [40], [88]–[90]. We found that the relative proportion of unsaturated FAs and the length of FA chains did not change significantly with cold acclimation in *D. melanogaster* larvae. The relative proportion of GPEtns was significantly lower in the 25°C- and 15°C-acclimated larvae than in the 15°C→6°C-acclimated larvae, which is in agreement with the generally expected trend. The ethanolamine moiety is less hydrated and occupies a smaller area than the choline moiety. As a result, GPEtns assume a more conical conformation and pack less efficiently into the lipid bilayers than GPChols, thus decreasing their order. Increase in the relative proportion of GPEtns in cold thus counteracts the ordering effects of low temperatures [91]. In this study, 50% of all the molecular species of GPLs were slightly but significantly influenced by cold acclimation. Most of the changes, however, were of so small a magnitude that it would be too speculative to explain them on an adaptive basis. We reached a similar conclusion in our previous analysis of membrane lipids in *D. melanogaster* adults [17]. One change, however, merits special attention. In both the larvae and the adults of *D. melanogaster*, the most prominent change related to cold acclimation was the increase in the relative proportion of GPEtns with linoleic acid (FA 18∶2) esterified at the *sn*-2 position of glycerol. In a study [41], the level of GPEtn 16∶0/18∶2 in *C. costata* larvae was also elevated by almost 20% during cold acclimation. A similar response has been reported in some other insects [52], [92]. It has been proposed that the adaptive value of specific pairing of palmitic and linoleic acids in a single GPL molecule may be related to the widening of the window between the temperatures of gel phase transition (*T_m_*) and hexagonal phase transition (*T_h_*), thus increasing the span of environmental temperatures at which the membrane remains fluid and functional [92]. Verification of this hypothesis requires further study.

Overgaard and coworkers observed very small changes in the FA composition of membrane GPLs in *D. melanogaster* in response to RCH [93], [94]. The response was characterized by an increase in the proportion of linoleic acid (FA 18∶2) at the expense of palmitic acid (FA 16∶0) and oleic acid (FA 18∶1). Typically, all the changes were in the order of less than 1 molar percent, but together they caused a slight increase in the proportion of the unsaturated FAs and a decrease in the average FA chain length. Slow cooling rates (*i.e.*, longer RCH) resulted in more pronounced changes, which indicated that membrane restructuring requires some time. Both slow and fast rates of RCH showed positive effects on survival after cold shock, despite that they differed in the absence and presence of membrane remodeling response, respectively. Influence of RCH on membrane remodeling was re-assessed later [25] and no significant effects of RCH on the molar percent composition of FAs or on the relative proportion of unsaturated FAs was found. These results suggest that membrane remodeling is not a significant mechanistic explanation of RCH response in *D. melanogaster*.

## Supporting Information

Dataset S1 **Basic physiological parameters of variously acclimated 3rd instar larvae of** ***Drosophila melanogaster*** **.**
(XLS)

Dataset S2 **Concentrations of metabolites in body fluids of variously acclimated 3rd instar larvae of** ***Drosophila melanogaster*** **.**
(XLS)

Dataset S3 **Relative proportions of phospholipids in membranes of variously acclimated 3rd instar larvae of** ***Drosophila melanogaster*** **.**
(XLS)
